# Reply to Schade G. Comment on Hess et al. “Assessing Agreement in Exposure Classifications between Proximity-Based Metrics and Air Monitoring Data in Epidemiology Studies of Unconventional Resource Development.”

**DOI:** 10.3390/ijerph17165801

**Published:** 2020-08-11

**Authors:** Judy Wendt Hess, Gerald Bachler, Fayaz Momin, Krystal Sexton

**Affiliations:** 1 Shell Health Risk Science Team, Shell Oil Company, 150 North Dairy Ashford, Houston, TX 77079, USA; fayaz.momin@shell.com (F.M.); krystal.sexton@shell.com (K.S.); 2 Shell Health Risk Science Team, Shell International B.V., Carel Van Bylandtlaan 16, 2596 HR The Hague, The Netherlands; g.bachler@hotmail.com

We appreciate the comments by Dr. Schade [1] and respond to each below. We would also encourage Dr. Schade and others to read our response [2] to an earlier comment on our paper [3] in which we pointed out, among other things, that 90% of subjects included in the epidemiology studies we critiqued lived far from shale development areas and likely had no exposure to air pollutants from these operations. This is problematic, given that subjects were categorized into exposure quartiles for the analyses, and likely explains why we found evidence of significant exposure misclassification.

We did not claim to have performed a validation of well-activity (WA) proximity models in our analysis. Since WA values are not estimates of ambient pollutant concentrations, this would not have been an appropriate analysis, and we were careful to point out this distinction. We did not assess whether continuous WA values and air pollutant concentrations were correlated but rather whether there was general agreement between *exposure classifications* based on WA and air pollutant concentrations. As we stated in our paper [4]: “The question we essentially asked was, if these monitoring sites were instead a sample of epidemiology study subjects’ homes with monitors placed outside the front door, how well does the categorization of exposure agree between the two methods?”.

Each of the pollutants included in our analysis were suggested in published epidemiology studies as a possible mechanism behind reported health impacts of unconventional resource development (URD). WA is not itself a biological actor, but from an air pathway perspective represents one or more ambient pollutants related to shale development that are assumed to be present in higher concentrations near well sites. This is again seen in two recent URD epidemiology studies:

“Air pollutants associated with [oil and gas development (OGD)] include…PM2.5, diesel PM, nitrogen oxides (NOx), secondary ozone formation, mercury, and volatile organic compounds (VOCs) like benzene, toluene, ethylbenzene and xylene (BTEX)” … “Several OGD-related environmental exposures have been linked to reduced birth weight and gestational age … e.g., PM2.5, NOx, SOx [5].”

“Previous study has found that oil and gas preproduction produces ambient air pollutants, including fine particulate matter, nitrous oxides, volatile organic compounds, ozone, carbon monoxide, and hydrogen sulfide.” … “The etiology of preterm birth is suspected to include dysregulated inflammation, which may be a response to infection or oxidative stress associated with air pollution, including particulates and nitrous oxides [6].”

Because these and other authors imply that these pollutants are part of the biological pathway connecting URD and reported health effects, it was relevant to test these assumptions in our analysis. If Dr. Schade believes that these chemicals are not biologically relevant, this is an argument we believe is best taken up with the authors of these papers.

Regarding our use of the weighted kappa statistic, we did not use this test to assess correlation between two continuous data sets, again, because WA values are not estimating ambient air pollutant concentrations. While typically applied in clinical settings to assess interrater reliability, the kappa statistic performed equally well in our analysis, where we determined how frequently exposure classifications agreed between two methods of assignment. This was the fundamental question addressed in our analysis, and we provided confidence intervals to indicate statistical significance. Further, as described by Sim and Wright [7], the weighted kappa additionally “attaches greater emphasis to large differences between ratings than to small differences” because “disagreement by 1 scale point is less serious than disagreement by 2 scale points.” We also note that results of the kappa analysis were consistent with data presented in Figures 7–10 in our paper [4], which show the degree of misalignment in exposure classifications between the two methods.

The state of Pennsylvania requires detailed reporting of emissions by the oil and gas industry, and these data are available to the public [8,9]. Reports and visualizations can be generated specific to facility type, emission source, pollutant, municipality and more, including trends over time [10]. To be clear, WA was not used in these epidemiology studies as a “proxy for environmental impacts”; it was used as a proxy for individual-level URD exposure of each case and control in the studies. WA values were calculated on an index date for each subject, and then the entire distribution was divided into quartiles that were ultimately used in the calculation of odds ratios.

As stated in the classic text *Modern Epidemiology* [11]: “the objective of an epidemiologic study is to obtain a valid and precise estimate of the frequency of a disease or of the effect of an exposure on the occurrence of a disease in the source population of the study.” Our analysis supports Dr. Schade’s assessment that WA appears to be a poor proxy for environmental exposures, which, in our view, leads to biased exposure categorizations and undermines confidence in the results of epidemiology studies that used them.
